# Supplementary material for: Are carbon emissions trading and green financial instruments synergistic? -Comprehensive quantitative research based on content analysis
Source: PLoS One. 2024 Mar 7;19(3):e0298601. doi: 10.1371/journal.pone.0298601 (PMC10919601; doi:10.1371/journal.pone.0298601)
Supplement: S1 Data — (ZIP) [file pone.0298601.s001.zip › Data/地级市Z_F工作报告.zip!/║╙─╧/╜╣╫≈╩╨/└┤╫╘╣1⁄2╓┌║┼í╛┬φ┐╦╩2╛▌═°í┐_2013.docx]

各位代表：

现在，我代表市人民政府向大会作工作报告，请予审议，并请各位政协委员和列席人员提出意见。

2012年工作回顾

一年来，面对严峻复杂的经济形势，在省委、省政府和市委的坚强领导下，在市人大、市政协的监督支持下，市政府深入贯彻落实科学发展观，抢抓中原经济区建设重大机遇，强力推进中原经济区经济转型示范市建设，全市经济社会保持了平稳较快发展的良好态势。地区生产总值1576.3亿元，增长11.3%，高于全国、全省平均水平3.5个、1.2个百分点。全口径财政收入160.9亿元，其中公共财政预算收入85.1亿元，增长14.3%；人均公共财政预算收入和人均公共财政预算支出分别达2414元、4718元，高于全省平均水平372元、144元。规模以上工业增加值910.3亿元，增长14.2%。全社会固定资产投资1154.2亿元，增长21.6%。社会消费品零售总额437.2亿元，增长15.4%。城镇居民人均可支配收入20136元，增长11.8%，高于全省平均水平2.3个百分点；农民人均纯收入10113元，增长13.6%，高于全省平均水平2.3个百分点。

（一）经济转型示范市建设开创新局面。围绕抓转型、促发展、做示范，制订了实施方案，出台了21个专项规划和工作方案，实施了十大专项行动计划。与29个省直部门和金融机构签订战略合作协议或建立厅市会商机制。经济转型示范市建设战略、11个重大产业基地和重要平台、23个重大项目列入国务院批复的《中原经济区规划》。我市成为国家资源综合利用“双百工程”首批示范基地、国家发展改革委全国经济转型发展工作联系点。争取上级资金81.3亿元。经济转型示范市建设已成为全市上下的共识和行动，取得了明显成效。

（二）重大项目建设实现新突破。29个省考核重点项目完成投资124.7亿元，占年度计划的177.9%，居全省第二位。436个市重点项目完成投资698.6亿元，占年度计划的167%；其中亿元以上项目275个，增加71个，完成投资580.9亿元，增长13.1%；10亿元以上项目47个，增加13个，完成投资219.9亿元，增长28.2%，省市重点项目均实现了计划新开工项目百分之百开工、计划竣工项目百分之百竣工、计划投资提前一个季度百分之百完成。中国兵装焦作装备产业园等项目开工建设，佰利联年产6万吨氯化法钛白粉等项目加快推进，焦作东500千伏输变电工程等项目建成投用，奇瑞汽车年产61万台发动机项目提前下线、批量生产，创造了新的焦作速度。

（三）工业转型升级取得新进展。及时出台促进经济平稳较快增长的意见，采取50条具体措施，有力保障了工业经济稳定增长。规模以上工业企业1076家，主营业务收入3629亿元，增长15%。优势产业稳定增长。六大战略支撑产业、四大新兴产业规模以上工业增加值分别增长12.1%、19.2%。承载能力不断增强。9个省级产业集聚区规划调整方案全部通过审批，通过率全省第一，新增规划面积62平方公里；规模以上工业主营业务收入1980.6亿元，增长33.4%；固定资产投资654.7亿元，增长47.8%。新区行政区划调整全部完成，公共财政预算收入增长40.6%，规模以上工业增加值增长22.2%，全社会固定资产投资增长27.9%。两化融合全面提速。创建省首批信息化和工业化融合试验区2个、示范企业10家。智慧焦作成功签约，数字焦作建设加快，无线城市完成投资5.1亿元。创新能力有效提升。新增省重大科技专项3项，总数达6项。新增国家级高新技术企业7家，总数达34家。新增院士工作站2家，总数达10家。新增省级工程技术研究中心14家，居全省第二位。国家级、省级企业技术中心分别达3家、71家，总数居全省第三位。节能减排成效显著。万元工业增加值能耗下降15.76%，预计化学需氧量、氨氮、二氧化硫、氮氧化物排放总量分别削减7％、8%、10%、12%。我市成为全省首家环境容量研究及运用试点市和总量减排试点市之一。

（四）农业现代化迈上新台阶。粮食实现九连增。总产204.8万吨，增产4.3万吨。夏粮单产511.1公斤，居全省第一；玉米高产创造两项全国新纪录。集群效应显现。面品、怀药、肉品、果蔬等九大农业产业化集群企业实现销售收入446.3亿元、利润46.2亿元、税收16.9亿元，分别增长15.8%、17.3%、16.1%。新增规模以上农产品加工企业23家，总数达293家。台湾农民创业园建设有序推进，现代农业示范区建设全面加快。组织化程度提升。新增农民专业合作社420家，总数达3208家，覆盖率达45.6%。新增农村土地经营权流转面积28.5万亩，总数达62万亩。农机装备水平领先。主要粮食生产综合机械化水平达96%，居全国前列。农业生产条件改善。完成汤庙等6座小型水库除险加固工程。治理水土流失面积40平方公里，发展节水灌溉面积9.95万亩。完成平原区7.3万人村村通建设任务，解决了10.5万农村居民饮水安全问题。实施扶贫开发整村推进项目94个，易地搬迁1个，脱贫2.23万人。连续14年实现耕地占补平衡。林业生态建设加快。国家森林城市创建全面启动，造林14.5万亩、森林抚育和改造4万亩，森林覆盖率达25.7%。

（五）第三产业发展呈现新繁荣。巩固提升文化旅游业。全面创建国际知名旅游城市，积极推进十大文化旅游园区建设，云台山成为全国旅游标准化示范单位和全国首批知名品牌创建示范区，云台山景区数字化技术应用规范上升为国家标准，影视城成功创建国家AAAA级景区。与晋城市和南水北调中线工程沿线13个城市签订旅游合作协议，与韩国庆州市结为旅游友好城市。我市成为世界旅游城市联合会发起城市和首批会员城市、省级旅游标准化试点城市。全市接待游客2605.2万人次，实现旅游综合收入200.3亿元，分别增长14.2%、16.5%。加快发展商贸服务业。扎实推进万村千乡市场工程，完成200家农家店信息化改造，家政服务网络加盟企业达1640家。全面提升金融服务业。金融机构各项贷款新增104.4亿元，增长18.2%。中小企业贷款新增37.5亿元，增长21.2%。获省级银行授信800亿元。全市直接融资131.4亿元，增长35.4%。中证期货、洛阳银行落户焦作，市商业银行与港中旅顺利完成战略合作。

（六）城乡一体化发展迈出新步伐。新型城镇化建设加快。编制完成中心城市组团式发展总体规划、城市近期建设规划和土地收购储备规划，城市建设三年大提升行动全面启动。交通体系日益健全。郑焦城际铁路交付线路96%，桃花峪黄河大桥主体桥面全线贯通，焦桐高速温巩段、武陟至云台山高速开工建设；建成干线公路170公里、农村公路266公里。成功争取世界银行绿色交通项目贷款1亿美元。南水北调工程进展顺利。总干渠桥梁等交叉工程施工范围和新月铁路改线工程征迁工作圆满完成，焦东路、南通路、文昌路、詹泗路等跨渠桥梁建成通车，塔南路、普济路恢复通行，供水配套工程开工建设，安置房建成137.87万平方米。城市功能更加完善。城市水系引黄入焦、大沙河治理、灵泉湖围堰工程开工建设，北部山区生态治理恢复取得阶段性成果，闫河公园建成开园，普济河、群英河改造顺利推进。环城快速通道开工建设，新华街等12条道路大修竣工。东方红广场人防工程主体封顶。改造老城区供水管网32公里、燃气管网51公里，中心城区燃气普及率达91%，新增供热面积60万平方米。城市管理水平不断提升。集中整治城区市容秩序，大力整治背街小巷和户外广告，规划建设专业市场，数字城管覆盖面持续扩大。新型农村社区建设扎实推进。投资41.9亿元，开工建设68个社区，建成住宅2万余套，入住4.3万人，节约土地4700余亩。

（七）深化改革开放显现新活力。大力开展招商引资。深入推进招商引资项目落实年活动，引进市外境内项目1177个，总投资1526.9亿元，增长13.6%，合同利用外来资金1438.5亿元，增长15.4%，实际利用境内外资金933.6亿元，增长26%。努力扩大对外贸易。建成了焦作海关，海关监管场所、物流园区、综合保税区工作有序推进。全市进出口总值22.2亿美元，出口15.1亿美元，均居全省第三位。着力深化各项改革。积极推进市直局属企业脱钩改制，焦作市粮食集团组建运营。核减事业单位64家，市直事业单位绩效工资全面实施。完成第十批行政审批项目清理。公共财政改革不断深化，我市成为全国依法行政依法理财示范点。深化人才体制改革，引进高层次人才1133人，增长4.5%。深化教育改革，我市成为国家义务教育均衡发展综合改革试点市、国家深化中小学教师职称制度改革扩大试点市。深化医药卫生体制改革，我市成为国家全科医生执业方式和服务模式改革试点市，6县市全部成为国家县级公立医院综合改革试点县市，零差率销售药品3.65亿元，“先住院、后结算”服务模式惠及群众8.3万人，受到国务院医改办、省政府医改办充分肯定。

（八）保障改善民生达到新水平。投资34.7亿元，完成十项重点民生工程建设。财政用于民生支出达119.1亿元，增支21亿元，占公共财政预算支出的71.6%，比2011年提高3.8个百分点。教育事业快速发展。新建改扩建幼儿园102所，道清中学改造完成并投入使用，100个农村中小学改造全面完成，我市荣获全国两基工作先进单位。全市实现多媒体班班通中小学达60%，我市成为全国首批教育信息化试点市。职教园区一期主体完工，我市在全省率先实现职教强县全覆盖，再次荣获全省职教攻坚先进市。卫生服务明显提升。市第二人民医院外科病房楼建成投用，焦煤集团中央医院综合楼主体完工，41家社区卫生服务中心完成标准化建设，乡村一体化管理机制和乡村医生养老保障机制进一步健全。文化体育事业不断进步。新建农家书屋592家，总数达1992家，实现行政村全覆盖。市科技馆、图书馆免费开放，国际太极拳文化交流中心暨全民健身中心体育场和体育馆主体完工，建成国民体质检测中心，成立河南省武术段位制太极拳考评中心和中国武术段位考评点，成功举办市十二届运动会。人口计生工作稳步推进。认真落实计生家庭各项奖励优惠政策，在全省率先实现免费孕前优生健康检查项目全覆盖，出生人口性别比趋于平衡，人口自然增长率5.39％。。就业再就业工作持续加强。城镇新增就业8.52万人，失业人员再就业2.89万人，困难人员就业1万余人，农村劳动力转移就业4.17万人。在全省率先实现人力资源社会保障服务站行政村全覆盖，我市成为全省统筹城乡就业创业试点市。社会保障能力进一步增强。城乡居民社会养老保险制度实现全覆盖，参保率达99.4%，居全省首位。提高城乡低保和五保供养标准，加强老龄工作，80岁以上老人全部纳入高龄补贴保障，补贴人数从670人扩大到6.3万人，补贴标准全国领先。保障性住房新开工16559套，发放廉租住房租金补贴797万元。住房公积金覆盖面达78.2%，归集额、支取额和增值收益分别增长14.6%、19.3%和69.1%。深入开展爱心助残活动，提升残疾人保障服务水平，我市残疾人运动员冯齐夺得十四届残奥会举重冠军。社会大局和谐稳定。深入创建平安焦作，积极推进社会稳定风险评估，加强应急管理，社会大局持续稳定，我市荣获全国法治创建先进城市。深入推行领导干部轮流值班接待群众来访制度，信访工作不断加强。全面做好安全工作，安全生产形势持续好转。健全食品安全监管体系，在全国地市级政府中首家设立食品安全突出贡献奖，食品安全工作经验得到国务院和省委、省政府肯定。在全省率先完成城市社区基层组织换届，和谐社区建设走在全国前列。军地军民关系和谐融洽，实现全国双拥模范城四连冠。

（九）政府自身建设得到新加强。政府性投资建设项目严格实行十个百分之百管理，审减项目资金12.1亿元，审减率8.7%。全市公共资源交易中心进场交易项目2290个，增效节支11.1亿元。推进依法行政，清理规范性文件544件，废止和失效87件。加强政风行风建设，深化岗位权力风险防控，在全省首家开通运行电子监察行政处罚系统，我市成为国家预防腐败局全国廉政风险防控工作联系点。开展公车治理，加强公务支出预警管理，三公经费总额连续三年零增长。全面公开市政府年度主要工作目标任务和完成情况，认真听取人大代表、政协委员意见，149件市人大代表建议和473件市政协委员提案全部办结。援疆工作进展顺利。统计调查、审计、工商、民族宗教、检验检疫、气象、地震、档案、广电、史志等工作都取得了新的成绩。

各位代表！2012年，我市经济社会在科学发展道路上迈出了新步伐、取得了新成绩，这是市委坚强领导的结果，是市人大、市政协、焦作军分区大力支持的结果，是全市人民共同奋斗的结果，在此，我代表市人民政府，表示衷心的感谢！

各位代表！近年来，我们在严峻复杂的经济形势下找到了建设经济转型示范市的发展路子，夯实了长远发展的基础，保障和改善了民生，全市经济实力明显增强，社会事业全面进步，人民生活水平显著提高。工作中我们深深体会到，发展是要务，要始终把科学发展摆到首要位置。思路是引领，思路正确就能事半功倍。项目是支撑，必须持续不断谋划建设大项目、好项目。开放是活力，一举应多变、一招求多效。改革是动力，勇于走向改革深水区。民生是根本，保障和改善民生是一切工作的根本出发点和落脚点。作风是保障，讲实话、出实招、办实事、求实效。这些经验凝聚着全市人民的心血、汗水和智慧，在今后工作中我们将毫不动摇地长期坚持。

各位代表！在肯定成绩的同时，我们也清醒地认识到，发展中还存在不少困难和问题，我们工作中还存在不足。一是新兴产业规模偏小，传统产业转型升级任务艰巨。二是资金、环境、土地等要素制约趋紧，促进资源集约节约利用、加强环境保护、建设生态文明日益迫切。三是创新意识不强，科技创新和成果转化能力有待提高。四是一些重要领域改革需要进一步解放思想，加大推进力度。五是解决中心城区交通问题迫在眉睫，老城区水电气暖等基础设施改造需要进一步加快，城市管理水平有待进一步提升。六是就业就学就医等民生工作需要进一步加大力度，改善困难群众生产生活、缩小收入差距等任务还很艰巨。七是一些同志重说不重做，作风不实，研究不透，标准不高，工作不力。八是少数干部讲排场、比阔气、奢侈浪费，一些领域腐败现象还很严重。对这些问题，我们将高度重视，严肃对待，采取有力措施，认真加以解决，决不能让这些问题继续存在下去，决不能让这些问题阻碍我们的发展。

2013年工作安排

2013年，是贯彻落实党的十八大精神的开局之年，是落实《中原经济区规划》、加快建设经济转型示范市、为全面建成小康社会奠定坚实基础的重要一年。今年工作的指导思想是：深入学习贯彻党的十八大精神，坚持以邓小平理论、“三个代表”重要思想、科学发展观为指导，全面贯彻落实中央、省委经济工作会议精神，抢抓机遇、开拓创新、加快转型、持续发展，在加快信息化进程中持续探索“两不三新”三化协调科学发展的路子，促进四化同步发展，努力提高经济增长的质量和效益，更加注重保障和改善民生，加快建设经济转型示范市，奋力走在中原经济区前列。

今年的主要目标是：地区生产总值增长11%；公共财政预算收入增长12%；规模以上工业增加值增长14%；全社会固定资产投资增长22%；社会消费品零售总额增长17%；外贸出口增长16%左右；实际利用外商直接投资增长11%；城镇居民人均可支配收入、农民人均纯收入增长11%以上；城镇新增就业6万人以上，城镇登记失业率控制在4.5%以内；人口自然增长率控制在6.5％。以内；节能减排完成省下达任务。

实现上述目标，必须坚持总要求、突出四重点。坚持总要求，就是强力推进经济转型示范市建设，在转型中发展，在发展中提升。突出四重点：一是突出抓开放，坚定不移实施开放带动主战略，加大招商引资力度，以开放促改革、促创新、促发展。二是突出抓城镇化，坚持新型城镇化引领，坚持中心城市带动，拓展新型工业化、信息化、农业现代化发展空间，促进四化同步发展。三是突出抓改革创新，积极推进体制机制改革，努力消除制约科学发展的障碍和瓶颈，激发各类市场主体的创造力，进一步释放改革红利。四是突出抓创造新优势，加快重大基础设施建设，为转型发展、可持续发展夯实基础，提供有力支撑。

重点抓好九项工作：

（一）强力推进改革开放。坚持把对外开放作为应对复杂局面和破解各种制约的综合性战略举措，推动对外开放向更宽领域、更高水平迈进。拓展开放空间。积极推动与郑州融合发展、与豫西北联动发展、与晋东南合作发展，加强经济、文化、科技、人才、旅游等方面交流合作，形成携手共进、发展双赢的新局面。扩大开放领域。积极支持外来资本投向高成长产业、战略新兴产业等领域，强力推进文化旅游、医疗卫生、教育科技、现代农业、商贸物流、金融保险、信息服务等领域对外开放，构建全方位开放格局。大力招商引资。围绕《中原经济区规划》和经济转型示范市建设，积极对接中国和世界500强企业，不断深化与中国兵装、中国兵器、华润集团、奇瑞汽车、河南煤化等企业的战略合作，延伸产业链条，培育产业集群。加强对外贸易。加快国家级皮毛出口基地、河南省汽车零部件出口基地建设，提升优势产业整体水平。争创一批省级出口食品农产品质量安全示范区，积极申报省级国际服务外包示范园区，进一步增强外贸服务能力。优化发展环境。开展项目服务年活动，完善招商服务机制，提升服务质量、服务效率；完善社会信用体系，健全守信激励、失信惩戒机制，切实维护投资者合法权益，努力创造最佳投资环境，让投资者在焦作创业安心、生活舒心、工作顺心。

加快重点领域和关键环节改革。深化企业改革，推进国有企业股份制、公司制改革，全面推开厂办大集体企业改革。认真落实中小微企业支持政策，促进非公有制经济加快发展。深化财政体制改革，加强财税征收管理，培植壮大高效财源，深化部门预算改革，提高财政科学化、精细化管理水平。深化农村改革，推进集体林权、国有林场和供销合作社体制改革，加快农村金融改革创新。深化事业单位分类改革，完成事业单位分类任务，深入推进文化、教育、水利、运管等事业单位改革。深化医药卫生体制改革，制订医改资金支持计划，加快县级公立医院综合改革，推进医保总额预付、按病种付费等支付制度改革，积极推行全科医生执业方式和服务模式改革，大力开展“先住院、后结算”等便民服务。深化人才机制创新，完善人才政策，健全人才培养引进、合理配置、激励保障等机制，启动人才服务产业园前期工作，大力引进高层次人才。深化土地管理创新，加快推进农村集体土地确权登记发证工作，扎实开展土地综合整治，稳妥推进人地挂钩试点，探索结余建设用地指标流转。深化投融资体制改革，发展直接融资，引导创业投资，培育规范风险投资和股权投资。规范发展五大投融资平台，提升融资能力，扩大融资规模。

（二）强力推进新型城镇化建设。大力实施14820城市转型工程，深入开展城市建设三年大提升行动，加快推进城乡发展一体化，打造宜居城市。促进新老城区协调发展。编制新区总体发展规划，完善新区功能，加快中原路、中南路、世纪路西延、文汇路等道路建设，高标准规划建设新河区域，力争三年内初步建成融高端商务、生态旅游、休闲娱乐于一体的现代城市综合功能复合区，强力打造城市发展新亮点。编制老城区提升规划，加快老城区改造，做好山阳路、普济路等大修工作，打通一批断头路，整修一批瓶颈路，大力发展公共交通，缓解交通拥堵状况。加强城市管理，完成76条背街小巷治理工作。改造老城区供水管网30公里，确保水质综合合格率达到99.5%以上。加快天然气利用二期工程建设，改造老城区燃气管网50公里。实施集中供热管网工程，新增供热面积40万平方米。加快污水处理厂搬迁，推进第二污水处理厂和污水管网建设。提升垃圾无害化处理水平。把北部山区纳入城市规划范围，严格规划控制。积极推进城中村改造。加快国道省道建设，新改建农村公路200公里。推进农业人口有序转移。增强产业支撑能力，增强教育、医疗等基本公共服务能力，完善户籍、土地、财税、社保等配套政策，有序推进农业转移人口市民化。稳妥推进新型农村社区建设。完成总体布局、土地利用、产业发展等规划，加快68个已开工社区建设，20个社区达到入住条件。

（三）强力推进新型工业化发展。实施工业产业转型升级年度行动计划，加快40个重大转型升级项目建设，努力构建结构合理、特色鲜明、节能环保、竞争力强的现代工业产业体系。加快重大项目建设。产业升级方面，重点推进奇瑞汽车年产61万台发动机（二期）、中国兵装焦作装备产业园等项目；科技创新方面，重点推进多氟多锂离子电池关键材料、卓立烫印热升华成像技术等项目；高新技术方面，重点推进中轴集团数控电动螺旋压力机等项目；老城区企业搬迁方面，重点推进昊华宇航老厂区搬迁改造等项目。加快新区产业集聚区建设。积极推进新区申报国家级高新区和创建国家级新型工业化示范基地，新区主要经济指标要努力走在全省城市新区前列。9个省级产业集聚区规模以上工业主营业务收入达到2200亿元，完成固定资产投资800亿元，进一步提升综合带动效应。培育壮大骨干企业。大力支持30强、30高企业发展，实施标准化引领、质量提升和品牌带动工程，增强核心竞争力，培育一批规模优势突出、经济效益显著、行业地位领先的重点骨干企业。推进信息化工业化融合发展。加快企业智能化改造，推进企业管理信息化，两化融合评测定级企业达到全市规模以上企业的30%。加强科技创新工作。推进河南理工大学科技园建设。新增国家级研发中心2家、国家级高新技术企业3家以上、省级研发中心10家，省级以上研发平台达到100家。

（四）强力推进新型农业现代化发展。始终把三农工作作为重中之重，积极推进农业科技创新，加大投入力度，构建农业新型经营体系，提升农业现代化水平。提高粮食生产能力。加快实施高标准粮田百千万工程，新增高标准粮田40万亩。加强小麦、玉米超高产新品种选育，组建大型种业集团。发展农业产业化集群。壮大面品、怀药、肉品、果蔬等九大农业产业化集群，实现销售收入500亿元以上。加快台湾农民创业园建设。推进主食产业化。规划建设主食产业化园区，建成20家主食便民连锁店；实施粮安工程，保障居民粮食供应。提升农机化水平。新增农机具5000台，农机总动力达到400万千瓦，主要粮食生产综合机械化水平保持在96%以上。加强农业基础设施。加快蟒河等8段中小河流治理，完成司家沟等7座病险水库除险加固工程。推进孤山湖水库建设。加快实施小农水项目，发展节水灌溉面积5万亩，治理水土流失面积40平方公里。全面完成三年内解决平原区村村通和山丘区群众吃水问题目标任务。提升农业组织化程度。发展新型农民合作组织，农民专业合作社达到3500家以上。促进农业规模经营。加快农村土地经营权流转，鼓励和支持承包土地向专业大户、家庭农场、农民合作社流转，力争土地流转面积达到90万亩。健全现代农业服务体系。加强农业科技、质量安全、市场信息、气象防灾减灾等服务体系建设，扩大基层农业技术推广、动物防疫和农产品质量监测体系覆盖面，提高农业社会化服务水平。

（五）强力推进现代服务业发展。加快发展文化旅游、商贸物流和金融服务业，推动现代服务业发展提速、总量扩张、结构优化。大力发展文化旅游业。围绕创建国际知名旅游城市目标，加快十大文化旅游园区建设，推进韩园、陈家沟争创国家AAAA级景区。加快云台山旅游股份有限公司上市步伐，强力开拓国内外客源市场。推进旅游信息化和标准化建设，做好国家级旅游服务综合标准化试点工作，创建全省旅游标准化示范城市。办好云台山国际旅游节。大力发展商贸物流业。加快商务中心区和特色商业区规划建设，扎实推进万村千乡市场工程、社区便民服务工程、放心早餐工程和农超对接惠民工程，推进物流园区建设，推动物流业与制造业联动发展。大力发展金融服务业。加大金融支持实体经济力度，重点支持重大项目建设、中小微企业发展和三农工作。推进企业上市和上市企业再融资，鼓励企业借助资本市场做大做强。加强金融风险防控体系建设，防范和化解金融风险。

（六）强力推进生态文明建设。把生态文明建设融入转型发展全过程，坚持节约资源和保护环境基本国策，推进绿色、循环、低碳、可持续发展。强化资源能源节约。大力推行节能降耗技术改造和公共领域节能，促进资源节约集约利用，降低资源能源消耗强度。加强环境保护。严格环保准入和污染物排放标准，强化工程减排、结构减排和管理减排。实施环境综合整治工程，加强水、大气、重金属、土壤污染防治和饮用水源地保护。实施中心城区扬尘、油烟、噪声、尾气污染综合治理，开展PM2.5监测工作。加强农村环境连片综合整治，控制农村面源污染。完善生态系统。加快建设城市水系，积极推进沁河、大沙河治理工程和引黄调蓄平原水库项目。加快北部山区公园建设，在中心城区建设游园20个，新增绿地面积60万平方米。深入开展国家森林城市创建，加快建设南太行生态景观带、高速公路和林邓线两侧绿化、环城森林公园体系、林区生态保护型人工增雨示范基地，力争完成造林和森林抚育16万亩。通过我们的辛勤努力，加快建设美丽焦作，下决心让焦作的天更蓝、水更清、人与自然更和谐。

（七）强力推进民生工作。继续实施十项重点民生工程。办好一批关系人民群众切身利益的事情。扩大城乡就业。加快建设全省统筹城乡就业创业试点市，做好高校毕业生、城镇就业困难群体和农村富余劳动力等就业工作，实现失业人员再就业1.9万人、困难人员就业6400人、农村劳动力转移就业4万人。努力增加居民收入。推进收入分配制度改革，完善职工工资正常增长机制，提高低收入职工、基层一线职工工资水平。稳步推进农业劳动力向非农产业转移，增加工资性收入。全面落实强农惠农富农政策，多渠道增加农民收入。进一步加大对口帮扶力度，帮助革命老区群众脱贫致富。完善社会保障体系。继续提高城乡低保标准、五保供养标准和优抚对象补助水平。积极推动城镇职工养老保险与城乡居民养老保险衔接，城镇职工医保与居民医保、新农合衔接。扩大住房公积金制度覆盖范围。新开工建设保障性住房16191套，缓解低收入家庭住房困难。积极推进残疾人社会保障和服务体系建设，发展社会福利和慈善事业。加强社会养老服务体系建设，推进老龄服务产业项目，大力发展老龄事业。加快发展教育事业。新建改扩建幼儿园100所，全面完成学前教育三年行动计划目标任务。推进国家义务教育均衡发展综合改革试点市建设，实施中小学提升工程，提高城区中小学校生均公用经费标准，完成100个农村中小学优化改造项目，实现中小学多媒体网络教学全覆盖。大力发展职业教育。推进焦作卫生医药学校并入河南理工大学，支持高等教育发展。提高人民群众健康水平。支持解放军九十一中心医院与地方医院合作发展。完成市全科医生临床培养基地暨市人民医院病房楼主体工程，加快市第二人民医院新区医院、市儿童医院病房楼等项目建设。完善政策机制，提高出生人口素质，积极应对人口老龄化，推进人口均衡型社会建设。健全体育公共服务体系，构建15分钟健身圈，办好国际太极拳交流大赛，做好省十二届运动会筹备工作。发展文化事业。开展舞台艺术送农民等文化下乡活动，完成山阳故城保护基础设施项目建设，做好第七批国家重点文物保护单位申报工作，推进太极拳申报世界非物质文化遗产。加强和创新社会管理。增强城乡社区服务功能，强化基层社会管理和服务体系建设。加强社会管理综合治理，提升应急管理水平，建设平安焦作。推进“六五”普法工作，建设法治焦作。坚持一岗双责、分级负责，加强安全生产工作，完善保障机制，防范重特大事故；认真落实食品安全十项制度，严惩重处食品安全违法犯罪行为；畅通和规范群众诉求表达、利益协调、权益保障渠道，认真做好信访工作。壮大志愿者队伍，发展志愿者事业。进一步巩固和发展平等团结互助和谐的社会主义民族关系，依法加强宗教事务管理。加强国防教育和国防后备力量建设，支持驻焦部队、民兵预备役建设，深入开展双拥共建，巩固军政军民团结，促进军民融合式发展。保障改善民生是政府的重要任务，我们将持续加大民生投入，持续做好民生工作，让老百姓更多更好地享受发展成果，努力提高人民群众的幸福指数。

（八）强力推进十大建设。2013年实施市重点项目500个（第一批），总投资2523亿元，当年完成投资612亿元。加快推进事关焦作长远发展的重大工程，增强基础支撑能力，构筑转型发展新优势，突出抓好十大建设，总投资831.7亿元，2013年完成投资274.3亿元。一是在铁路建设方面抓好郑焦城际铁路、焦作东站升级改造、月山车站更名焦作西站、郑州到太原快速铁路通道焦作至晋城段（前期）等项目，总投资180.1亿元，2013年完成投资26亿元。郑焦城际铁路完成投资23亿元，焦作东站升级改造完成投资3亿元。二是在公路建设方面抓好焦桐高速温巩段、武陟至云台山高速、桃花峪黄河大桥、环城快速通道、迎宾路南延及焦作黄河大桥等项目，总投资146.9亿元，2013年完成投资26.1亿元。加快建设焦桐高速温巩段、武陟至云台山高速，建成桃花峪黄河大桥，环城快速通道力争竣工通车，迎宾路南延及焦作黄河大桥项目取得实质性进展。三是在能源建设方面抓好焦作电厂上大压小异地扩建工程、龙源电厂、焦作东区热电机组和城区电网改造等项目，总投资130.2亿元，2013年完成投资44.8亿元。焦作电厂上大压小异地扩建工程主厂房等全面开工，龙源电厂主厂房封顶、设备开始安装，焦作东区热电机组投入运行，城区电网改造全面启动。四是在水利建设方面抓好城市水系，总投资66.9亿元，建设北部山区影视湖、龙寺水库、圆融水库和新区灵泉湖、东湖五个水库，与新河、大沙河、蒋沟河贯通，形成贯穿城区东西总长48公里的水体景观；改造老城区白马门河、普济河、群英河、翁涧河、李河和山门河，形成贯穿城区南北总长50公里的城市带状公园，与南水北调河道形成“六纵四横”城市水网，解决城市地下水补源，改善区域气候和生态质量。2013年完成投资22.8亿元，全面完成引黄入焦总干渠工程，6月底前黄河水进入大沙河；年底前灵泉湖形成水面1000亩以上；积极推进引黄灌区项目前期工作，规划灌溉面积50万亩以上；实施水生态修复工程，打造大沙河带状湿地景观；年底前完成南水北调供水配套工程45公里输水线路建设任务，加快推进配套水厂建设。制订资金保障计划，确保城市水系2015年初步建成，一个泛舟河上、人水相依的宜居之城，一个碧波荡漾、河清水秀的中原明珠，一个水在城中、城在水中的新焦作将展现在我们面前。五是在生态建设方面抓好南水北调城区段绿化景观带工程、北部山区生态治理恢复，总投资37.3亿元，2013年完成投资14.1亿元。投资12.4亿元，开工建设南水北调城区段绿化景观带工程安置房73.32万平方米；投资1.7亿元，持续推进北部山区生态治理恢复。六是在信息化方面抓好无线城市和智慧焦作，总投资10.1亿元，2013年完成投资8.8亿元。无线城市完成投资6.7亿元，建设网络基础设施、民生信息化示范工程等；智慧焦作完成投资2.1亿元，建设市民一卡通、智慧政务、智慧旅游等。七是在发展载体方面抓好新区和产业集聚区10亿元以上项目，总投资240亿元，2013年完成投资120亿元。每个产业集聚区至少开工建设2个投资10亿元以上工业项目，每个城区至少开工建设2个投资10亿元以上商贸或工业项目，焦作新区至少开工建设4个投资10亿元以上工业或商贸项目，新开工项目当年完成投资50%以上。八是在教育事业方面抓好职教园区，总投资6.7亿元，2013年完成投资4.2亿元，完成职教园区一期工程建设，启动市技师学院新校区项目。九是在文化体育事业方面抓好国际太极拳文化交流中心暨全民健身中心，总投资13亿元，2013年完成投资7亿元，体育场馆基本建成。十是在开放型经济方面抓好焦作海关及综合保税区、物流园区，2013年完成投资4500万元。积极申报综合保税区，建成海关监管场所，焦作海关开关运行。十大建设关系我市长远发展，关系广大人民群众切身利益，是焦作转型发展、可持续发展的重要基础和有力支撑，是人民生活幸福的热切期盼和美好愿景，我们将制订专项行动计划，健全推进机制，加快实施，早日建成，造福焦作人民。

（九）强力推进政府自身建设。人民政府为人民。坚持问政于民、问需于民、问计于民，维护和发展好人民群众的根本利益，不断提高人民群众的舒适度和满意度，努力建设人民满意的政府。一是以人为本，建设为民政府。人民在我们的心中有多重、我们在人民的心中就有多重。想问题、办事情要把人民利益放到第一位，以人民满意为第一标准，真实了解群众愿望，真诚回应群众呼声，真情关心群众疾苦，解决好群众生活中遇到的实际困难，尤其要安排好困难群众、弱势群体、下岗职工的生产生活，让他们感受到社会主义大家庭的温暖。二是转变作风，建设务实政府。牢固树立实干兴邦理念，认真落实中央八项规定和省市意见，切实改进政风、文风、会风。坚持言必行、行必果，对确定的重大决策部署、重点工作任务，严格落实到位。坚持勇于担当、勇于负责，遇到矛盾不回避，碰到问题不推拖，敢于触及矛盾，敢于解决问题。坚持求真务实、真抓实干，少开会、开短会，少发文、发短文，坚决反对官僚主义、形式主义，真正把功夫用在推进发展、造福百姓上。健全工作考核奖惩机制，坚决不让老实干事的干部吃亏，坚决不让只说不做的干部混下去。三是履职尽责，建设效能政府。在其位谋其政，任其职尽其责，进一步增强慢不得的紧迫感、等不得的使命感、拖不得的责任感，时不我待、只争朝夕，多干利长远的实事，多办惠民生的好事，努力追求真金白银的发展、群众满意的实绩。健全决策机制和程序，提高政府决策的科学化、民主化水平。深化行政审批制度改革，提高行政审批效率。切实加强执行力建设，加大网络问政力度，奖优、治庸、罚劣相结合，提高行政效能，今日事今日毕，在执行上比速度，在服务上见高低，在推进工作上求实效。四是依法行政，建设法治政府。自觉接受人大、政协的监督，认真办理人大代表建议、政协委员提案，虚心听取各民主党派、无党派人士和人民团体意见，规范提升市政府与市人大代表、政协委员网络联系平台服务水平，做到事事有回音、件件有结果。深化法治型政府建设，规范行政处罚自由裁量权，畅通行政复议渠道，严格按照法定权限和程序行使权力。深入推进政务公开，创造条件、支持欢迎人民群众和新闻媒体监督政府工作，让权力在阳光下运行。五是清廉自律，建设廉洁政府。完善惩治和预防腐败体系建设，深化岗位权力风险防控，强化政府性投资建设项目十个百分之百管理，健全公共资源交易体系，更加注重从源头上预防和治理腐败，严厉查处权力寻租、权钱交易等违纪违法行为。坚持厉行节约，勤俭办一切事情，坚决管好钱袋子，坚决降低行政成本，确保三公经费下降15%以上，以为民、务实、清廉的良好形象，创造无愧于人民的崭新业绩！

各位代表!

实干铸就辉煌！让我们紧密团结在以习近平同志为总书记的党中央周围，在党的十八大精神指引下，在省委、省政府和市委的坚强领导下，凝心聚力，务实重干，加快建设经济转型示范市，奋力走在中原经济区前列，为全面建成小康社会而努力奋斗！
